# Molecular Wiring in Smart Dressings: Opening a New Route to Monitoring Wound pH

**DOI:** 10.3390/healthcare3030466

**Published:** 2015-06-25

**Authors:** Anna McLister, James Davis

**Affiliations:** School of Engineering, Ulster University, Jordanstown BT37 0QB, UK; E-Mail: mclister-a@email.ulster.ac.uk

**Keywords:** pH, chronic wounds, sensor, electrochemical, tryptophan

## Abstract

It has been proposed that fluctuations in wound pH can give valuable insights into the healing processes in chronic wounds, but acquiring such data can be a technological challenge especially where there is little sample available. Developments in voltammetric pH sensing have opened up new avenues for the design of probes that can function in ultra-small volumes and can be inherently disposable but, as yet few can meet the demands of wound monitoring. A preliminary investigation of the pH response of a new redox wire prepared from a peptide homopolymer of tryptophan is presented and its potential applicability as a sensing material for use in smart dressings is critically discussed.

## 1. Introduction

In recent years there has been a tremendous increase in the number and variety of wound dressings but, despite quite remarkable advances in material design and clinical procedures, the management of chronic wounds remains problematic. The treatment of diabetic foot ulcers (DFU), in particular, has long been a concern for healthcare providers; especially in light of the increasing prevalence of diabetes and the fact that 25% of those patients will be expected to encounter a DFU at some point in their lifetime [1]_._ Moreover, it has been estimated that the number of patients, worldwide, will rise to half a billion by 2030 [2], placing a severe burden on already stretched resources [3]. In an increasingly cost conscious health care system, there is a clear demand for a low cost system that is capable of monitoring the environment of the wound to aid clinical decision-making but, ideally, as an early warning system for the onset of potential complications to healing—particularly infection [4,5,6]. The latter is a critical factor which, all too often, can go undetected with intervention occurring only after the presentation of gross symptoms. Clinical assessment of chronic wounds is still fraught with ambiguity and heavily reliant on a number of variables such as reporting time, clinician experience, *etc.* At present, there is a significant drive to develop technologies that can be used at the point of care, within the clinic, but also within the home as part of a connected health outpatient strategy [7,8]. The latter is a considerable challenge in terms of designing smart materials that can function as the interface between the wound and the electronics. The design of sensor systems that can be integrated within wound dressings is a highly active area and one which is fraught with problems: biocompatibility, selectivity, sensitivity and sensor lifespan being only a few. The aim of this communication has been to investigate the bioelectrochemical sensing capabilities of a new material based on a homopolymer of tryptophan and to assess its potential use as a smart interface.

**Figure 1:**
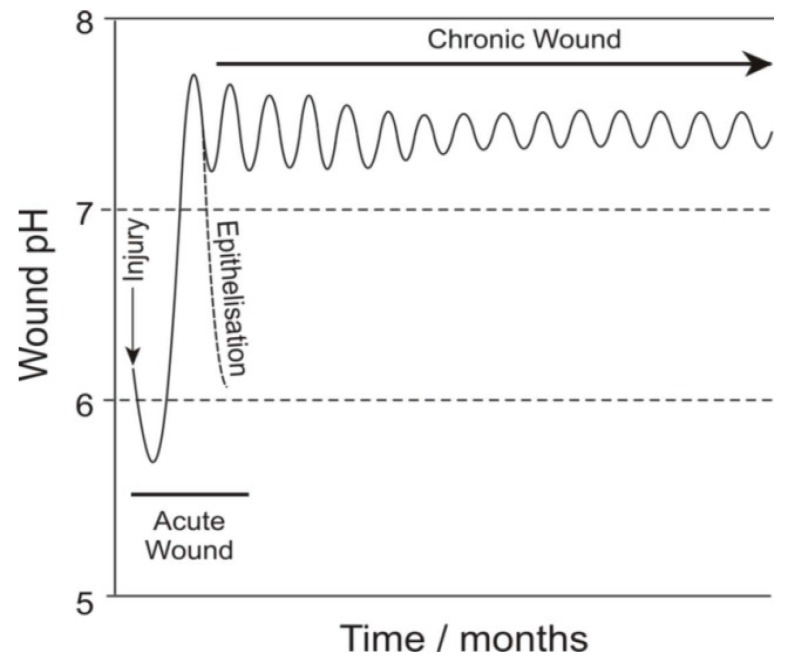
pH response dynamics for chronic and acute wounds. Adapted from Schneider *et al.* (2007) [9].

Wound monitoring can take a number of forms, therefore, examining both physical (temperature, moisture) and chemical markers and many of the detection strategies have been reviewed [7,8]. The use of molecular markers is arguably a more sophisticated approach in that the dynamics of particular biochemical species can give insights into different aspects of the wound status. In this work, the periodic monitoring of pH is considered as there is a significant amount of literature to support the relationship between the pH of a wound and its healing progress [9,10,11]. The normal pH of the skin is somewhat slightly acidic, with a pH typically in the region 5.5–6. However upon injury and the exposure of underlying tissue, the wound pH gradually increases to the normal homeostatic level of pH 7.4. As healing progresses, the wound pH will return to its normal acidic mantle. Unfortunately, in the case of a chronic wound, this healing process is stalled and the pH tends to oscillate in a narrow region between neutral and alkaline (Figure 1) and can remain there for a prolonged period that may cover many months [9].

The prolonged exposure to a higher pH creates a more favourable environment for bacteria, which in turn leaves the patient more susceptible to infection. The precise relationship between pH and infection however is far from clear and there is a need for more advanced tools that can monitor the changes *in vivo*—not simply for infection detection but possibly also for infection prevention. On a simple level, the oscillations can be used to represent a baseline and it could be envisaged that deviation beyond pre-set limits could be used as a signal to alert the patient. Colonisation of the wound by bacteria is inevitable but the change to an infectious/virulent state can be accompanied by a change in pH as the bacteria multiply or biofilm formation occurs at the sensor surface [9].

It is clear that the existing pH probe technology is far for suitable for use directly in assessing the pH of wound fluid but there have been considerable developments in recent years with a host of new approaches coming to the fore, largely in response to the limitations of the traditional glass potentiometric systems [7,8,12]. It must be noted that not all are designed specifically for biomedical contexts, nor indeed are they all appropriate. The implementation of sensor systems within the latter gives rise to numerous concerns where issues over probe size and disposability can be problematic—especially where sample sizes may be limited (as in the case of clinic DFU sampling) or *in vivo* application is desired. A number of research avenues have been explored to counter these issues and both potentiometric [12,13,14,15,16,17,18,19,20,21,22,23,24,25] and voltammetric [26,27,28,29,30,31,32,33,34,35,36,37] methodologies have been pursued. In most cases, a functional material containing pH sensitive components is applied to the surface of an appropriate sensor substrate through a variety of methods that include: Adsorption/monolayer [20,29], polymer films [17,34], screen printed inks [16,19,21,28], covalent attachment [27,28] or electrodeposition [13,14,18,23,24,25,33]. While such modifications can be useful for investigative purposes, there are obvious issues over electrode fouling and biocompatibility. The latter is particularly worrisome where there is the possibility of the pH sensitive component leaching into the biofluid.

Voltammetric approaches have only recently been adapted for the measurement of pH [26,27,28,29,30,31,32,33,34,35,36,37]. The approach taken has typically exploited the pH dependence of a quinone-hydroquinone (Q/H_2_Q) redox couple in which the position of either the oxidation or reduction peak is related to the pH of the fluid in accordance with the established Nernstian relationship of −0.059 m/n V/pH (where m/n is the ratio of protons to electrons) [26,27,28,29,30,31,32,33,34,35,36,37]. The aim of this communication has been to assess an alternative approach wherein the pH sensitive quinone redox groups are first generated *in situ* and are locked directly within the core structure of the polypeptide polymer of tryptophan.

**Figure 2:**
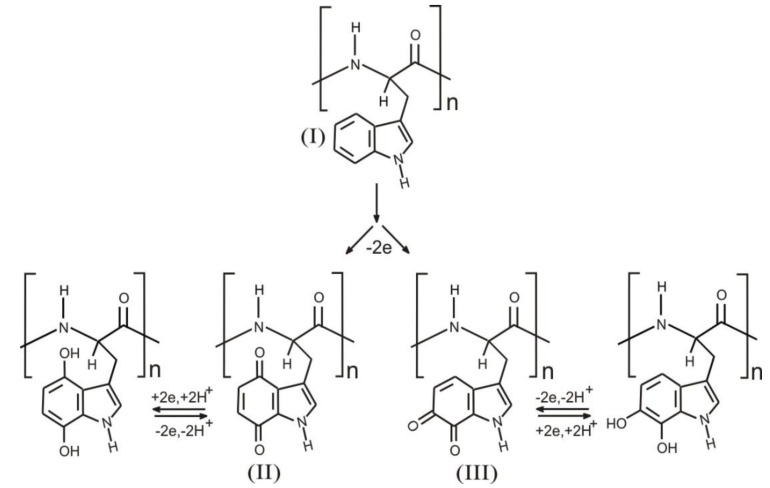
Electrochemical oxidation of polymer bound tryptophan to the corresponding quinone systems and their corresponding hydroquinone redox system.

The electrochemical properties of tryptophan are well established and there is an extensive literature base dedicated to the detection of the amino acid within a large variety of biological samples. It has recently been shown that upon oxidation, quinone groups are formed on the indole ring and that these exhibit pH dependent electrode responses [38]. The core rationale of the present investigation was to determine if these quinoid moieties can be formed within a polypeptide containing tryptophan and therefore exploited as a pH sensitive redox wire. The reaction scheme is shown in Figure 2 and involves the electro-oxidation of the indole components within the poly tryptophan (I); resulting in the generation of 1,4 (II); and 1,2 (III) quinoid species.

**Figure 3:**
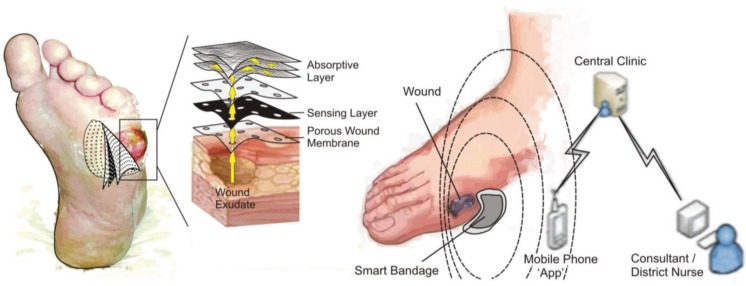
The proposed implementation and operation of a smart bandage based on a carbon fibre sensing layer onto which polytryptophan had been immobilized.

Due to the relatively short lifespan of any bandage, the challenge at hand is to develop a cheap and disposable sensor platform. On this basis, an innovative approach was taken through incorporating poly-l-tryptophan with a carbon fibre mesh resulting in the formation of a conductive composite. Carbon fibre has been used in countless sensor applications and has found application in wound diagnostics [8]. It was envisaged that the highly conductive nature of the fibre would enable electrochemical interrogation of the tryptophan polypeptide. The proposed implementation of the bandage is shown in Figure 3.

There has been substantial progress in the development of miniaturized potentiostats [39,40,41] and several have been specifically design for application to wound monitoring [40,41].

## 2. Experimental Section

Unless stated otherwise, the electrochemical measurements were conducted at 22 °C ± 2 °C in Britton Robinson buffer (acetic, boric and phosphoric acids—each at a concentration of 0.04 M and adjusted to the appropriate pH through the addition of sodium hydroxide). Electrochemical measurements were conducted using an Autolab PGStat computer controlled potentiostat (Eco-Chemie, Utrecht, The Netherlands). Initial investigations used a three electrode configuration consisting of a carbon fiber working electrode (encased in a polyester laminate sheath with a 16 mm^2^ window), a platinum wire counter and a 3 M NaCl Ag|AgCl half-cell reference electrode (BAS Technicol, Bristol, UK). The carbon fiber film was modified by drop casting poly-l-tryptophan (MW 15,000–50,000, Sigma-Aldrich, Gillingham, UK) that was dissolved in acetonitrile (5 mg/mL) onto the surface. X-ray photoelectron spectroscopy (XPS) of the pre-laminated carbon fibre surface before and after anodisation was performed using an Axis Ultra DLD spectrometer (Kratos Analytical, Hadano, Japan) using monochromated Al Ja X-rays (15 kV and 10 mA) with an operating pressure lower than 6 × 10^−8^ Pa. A hybrid lens mode was used during analysis and charge neutralisation was achieved using an immersion lens with a filament current of between 1.7 and 2.1 mA at a charge balance voltage of between 3.0 and 3.6 V. Three spots were analysed per sample and wide energy survey scans (0–1300 eV binding energy) as well as high resolution spectra for C_1s_ (272.5–297.5 eV) and O_1s_ (519.5–544.5 eV). Pass-energy was 160 eV for the wide energy survey scans and 20 eV for the high resolution spectra. Quantification of the atomic % oxygen at the surface from the high resolution spectra was conducted by subtracting a linear background and calculating the area under the peaks on the C_1s_ and O_1s_ spectra using Vision 2.2.8 software (Kratos Analytical).

## 3. Results and Discussion

**Figure 4:**
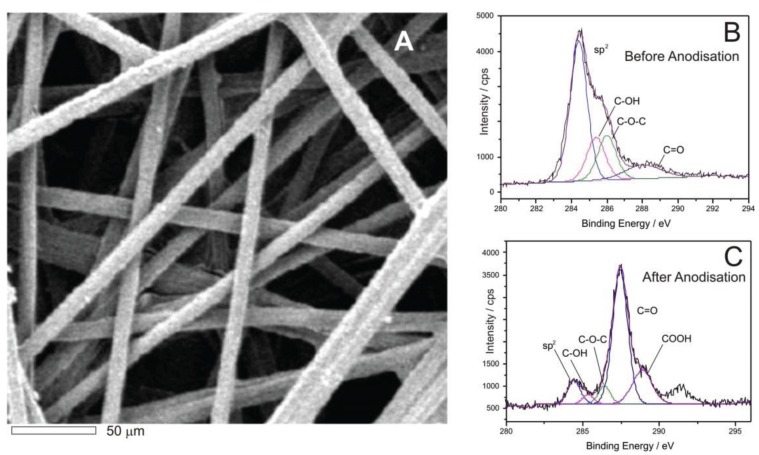
Scanning electron micrograph of the carbon fibre mesh (**A**); and XPS spectra obtained before (**B**); and after (**C**) electrochemical anodisation [42].

The structure of the carbon fibre mesh is detailed in the electron micrograph shown in Figure 4A and it can be observed that there is a relatively open network of fibres with a typical diameter of 10 micron. Although the fibre is conductive, previous work by the authors have demonstrated that the electrode performance of carbon substrates can be significantly enhanced through electrochemical anodisation [33,36,42]. Electron transfer can be relatively slow at carbon which is predominantly basal plane in structure and thus oxidation in alkali (+2 V, 0.1 M NaOH, 60 s) results in the partial exfoliation of the fibres and increases both the number of edge plane sites oxygen functional groups [42]. The degree of modification was confirmed through XPS analysis of the surface pre and post anodisation and with the spectra detailed in Figure 4B,C respectively [42]. A significant decrease in sp^2^ carbon (associated with basal structures) and an increase in carbonyl and carboxyl functionalities was found after the anodisation process. The poly-tryptophan solution was subsequently drop cast on to the anodisation fibre framework and this approach was utilized in all subsequent experiments.

Note that for the sake of brevity, that the 1,4-quinoid form is used as a representative structure of the oxidized tryptophan structure but that the heterogeneous nature of the electrochemical formation will also produce 1,2 quinone and other moieties [38].

**Figure 5:**
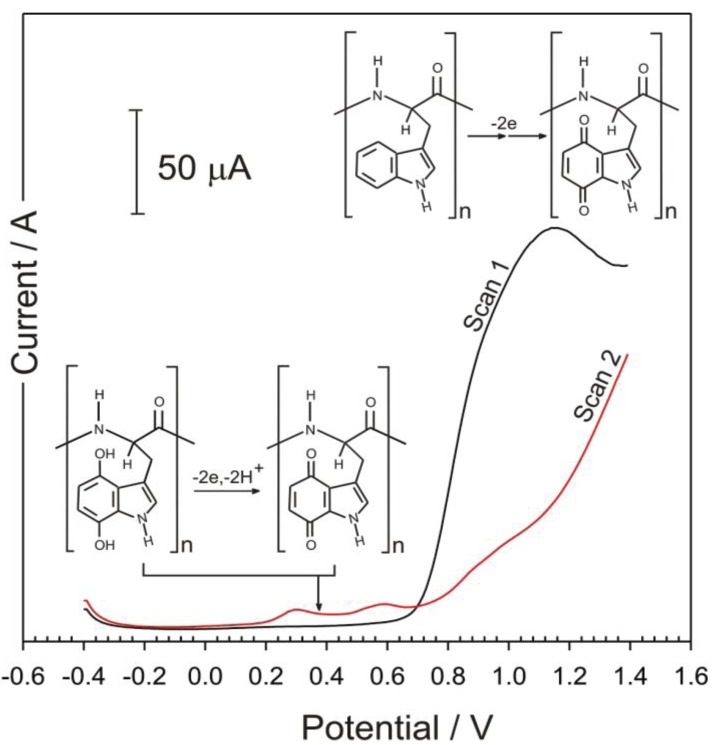
Square wave voltammograms detailing the first and second scans of the poly-tryptophan carbon fibre composite sensor.

The electrochemical properties poly-tryptophan modified carbon fibre composite were investigated principally using square wave voltammetry. Voltammograms detailing the first two, consecutive scans of the poly-tryptophan modified carbon fibre in pH 3 buffer are shown in Figure 5. Upon initiating the first scan, it can be seen that oxidation of the indole component occurs with the resultant peak observed at +1.2 V. The second scan reveals two new peak processes (+0.31 and +0.58 V) which correspond to the electrogenerated quinone processes and is in agreement with the monomer results previously reported where the two peak processes are attributed to the formation of 1,2 and 1,4 quinoid isomers [38]. The oxidation process associated with the original indole component is substantially diminished and can be attributed to the fact that the majority of the indole substituents, accessible to the underlying electrode, have been oxidized. There is a considerable disparity in the magnitude of the parent indole oxidation and the resulting quinone daughter peaks, also found previously [38]. It could have been anticipated that had the target molecule been monomeric tryptophan, some of the electrogenerated products may have been lost through diffusion away from the electrode surface. Given that in this case the target is locked into the insoluble polypeptide back bone, such an explanation is unlikely. It is clear that the initial oxidation of the indole is not a well-defined process and it has been reported that some 20 distinct products were found after the controlled oxidation of indole acetic acid [38]. It would appear that a similar product spectrum arises with the poly-tryptophan system used here. Nevertheless, the resulting daughter peaks are clearly resolved and, potentially, useful for diagnostic purposes.

**Figure 6:**
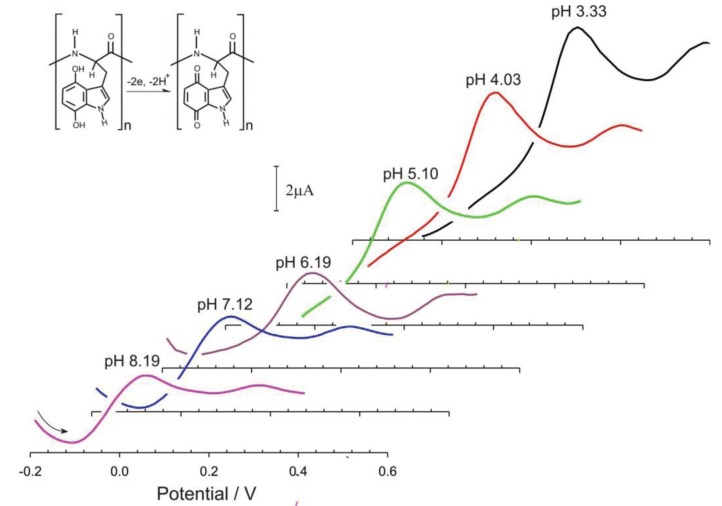
Square wave voltammograms detailing the influence of solution pH on the response of the poly-tryptophan—carbon fibre composite electrode.

The pH response of the electrogenerated indolic quinone moieties was also assessed using square wave voltammetry. The electrode was rinsed after the initial oxidation and placed in fresh pH 3 buffer. The pH was then manipulated through the drop wise addition of sodium hydroxide and the response of the carbon-poly-tryptophan mesh recorded in a dynamic fashion to highlight the ability of the polytryptophan to respond to fluctuations in pH. The response profiles are shown in Figure 6.

The main rationale behind using poly-l-tryptophan was on the basis that the peak positions of the electrogenerated quinoid functionalities would be pH responsive. This was postulated on the fact that there is a 2 electron, 2 proton transition between the oxidized and reduced components as detailed in Figure 2. Therefore it could be envisaged that the peak potential will shift by −0.059 V per pH unit in accordance with previously published voltammetric pH sensor data [26,27,28,29,30,31,32,33,34,35,36,37,38,42]. The voltammograms shown in Figure 6 certainly shift with pH, however the relationship is sub Nernstian (Epa (V) = −0.051 pH + 0.462; *n* = 6; *R*^2^ = 0.993) over pH 3 to pH 8. This can be ascribed in part to the slight broadening of the peak at higher pH.

**Figure 7:**
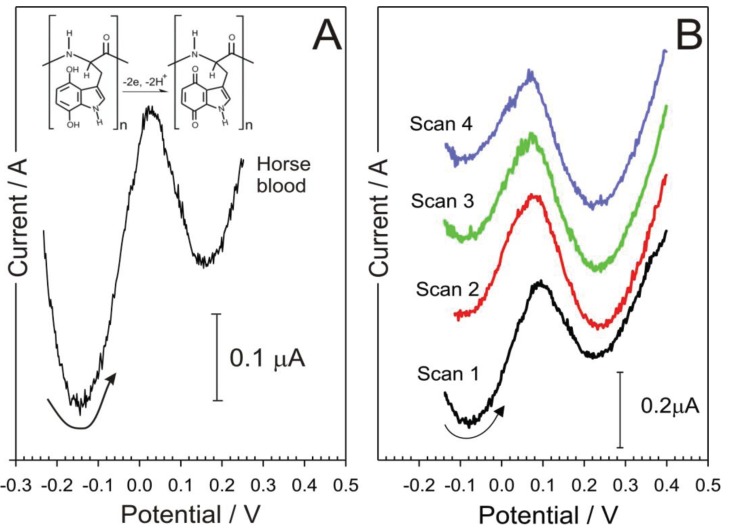
Square wave voltammograms detailing the electrode response to horse blood (**A**); and consecutive scans (**B**).

In a typical wound environment the sensor would be exposed to a variation of complex biofluids, for example blood. To determine the viability of the sensor response in a simulated wound environment, further testing of the sensor was carried out using horse blood. Horse blood was selected on the basis that it presents a compositionally complex fluid—containing mono and macromolecular species and cellular components—which could interact with the poly-tryptophan signal. Results from testing the prototype electrode in horse blood can be seen in the voltammograms detailed in Figure 7. The oxidation peak of the quinoid component within the poly-tryptophan film can be clearly seen. To further confirm that the peak formed was not simply due to the oxidation of other components found in blood (ascorbate, urate, *etc.*) [36,37], the response to a carbon fibre electrode without the poly tryptophan was recorded and found to be devoid of any competing processes within the potential range highlighted in Figure 7. The pH of the horse blood was assessed by examining the peak position of the quinoid oxidation (+0.082 V) and then inserted into the calibration equation noted earlier—much in the same way a conventional pH probe will measure an unknown and refer the potential to the internal calibration equation. In this case, the sample was found to be pH 7.45.

Consecutive scans (*n* = 4) revealed only minor variations in the peak position (±5 mV) but, given the log relationship, this can translate to ±0.1 pH units. The latter is encouraging and certainly within the accuracy needed to monitor fluctuations in the chronic wound pH oscillation indicated in Figure 1. The variation can be due to interaction of the quinoid constituents with other components within the blood sample.

## 4. Conclusions

Poly-tryptophan is an incredibly hydrophobic polymer that, hitherto, has found few applications and remained largely a curiosity. The tryptophan component itself is an incredibly versatile substituent and, as demonstrated, can enable the conversion of the passive polymer into a redox wire capable of monitoring pH. Although preliminary in nature, the results obtained highlight a wholly new route through which voltammetric pH sensing methods could be explored. The use of the tryptophan homopolymer is but a model system and it could be envisaged that other, more tailored, peptides, containing defined sequences of tryptophan units, could be used as reporter molecules and may go some way to creating sensing interfaces that are more biocompatible.
